# Feasibility of deep-inspiration breath-hold PET/CT with short-time acquisition: detectability for pulmonary lesions compared with respiratory-gated PET/CT

**DOI:** 10.1007/s12149-013-0774-9

**Published:** 2013-10-23

**Authors:** Shozo Yamashita, Kunihiko Yokoyama, Masahisa Onoguchi, Haruki Yamamoto, Shigeaki Hiko, Akihiro Horita, Kenichi Nakajima

**Affiliations:** 1 Division of Radiology, Public Central Hospital of Matto Ishikawa, 3-8 Kuramitsu, Hakusan, Ishikawa 924-8588 Japan; 2 Department of Health Sciences, Graduate School of Medical Sciences, Kanazawa University, 5-11-80 Kodatsuno, Kanazawa, Ishikawa 920-0942 Japan; 3 PET Imaging Center, Public Central Hospital of Matto Ishikawa, 3-8 Kuramitsu, Hakusan, Ishikawa 924-8588 Japan; 4 Department of Nuclear Medicine, Kanazawa University Hospital, 13-1 Takara-machi, Kanazawa, Ishikawa 920-8641 Japan

**Keywords:** Deep-inspiration breath-hold (DIBH) PET/CT, Respiratory-gated (RG) PET/CT, Pulmonary lesion, Reconstruction parameters

## Abstract

**Objectives:**

Deep-inspiration breath-hold (DIBH) PET/CT with short-time acquisition and respiratory-gated (RG) PET/CT are performed for pulmonary lesions to reduce the respiratory motion artifacts, and to obtain more accurate standardized uptake value (SUV). DIBH PET/CT demonstrates significant advantages in terms of rapid examination, good quality of CT images and low radiation exposure. On the other hand, the image quality of DIBH PET is generally inferior to that of RG PET because of short-time acquisition resulting in poor signal-to-noise ratio. In this study, RG PET has been regarded as a gold standard, and its detectability between DIBH and RG PET studies was compared using each of the most optimal reconstruction parameters.

**Methods:**

In the phantom study, the most optimal reconstruction parameters for DIBH and RG PET were determined. In the clinical study, 19 cases were examined using each of the most optimal reconstruction parameters.

**Results:**

In the phantom study, the most optimal reconstruction parameters for DIBH and RG PET were different. Reconstruction parameters of DIBH PET could be obtained by reducing the number of subsets for those of RG PET in the state of fixing the number of iterations. In the clinical study, high correlation in the maximum SUV was observed between DIBH and RG PET studies. The clinical result was consistent with that of the phantom study surrounded by air since most of the lesions were located in the low pulmonary radioactivity.

**Conclusion:**

DIBH PET/CT may be the most practical method which can be the first choice to reduce respiratory motion artifacts if the detectability of DIBH PET is equivalent with that of RG PET. Although DIBH PET may have limitations in suboptimal signal-to-noise ratio, most of the lesions surrounded by low background radioactivity could provide nearly equivalent image quality between DIBH and RG PET studies when each of the most optimal reconstruction parameters was used.

## Introduction

Positron emission tomography/computed tomography (PET/CT) with ^18^F-fluorodeoxyglucose (^18^F-FDG) can visualize human glycometabolism, and is widely used for the diagnosis of lesions and staging of diseases [1–3]. PET/CT can provide more accurate anatomical locations than dedicated PET system. It is, moreover, advantageous for shortening an examination time over PET, and attenuation is accurately corrected using μ-map calculated by Hounsfield units from CT images [4]. Recently, PET/CT is also used for radiotherapy and to assess the effectiveness of therapy [5–8]. However, misregistration between PET and CT images may occur because PET and CT data are acquired sequentially, and lesions detected by PET are not consistent with those detected by CT if the lesion is moved by the body motion, respiration and peristalsis. The motions result in unclear images and inaccurate standardized uptake value (SUV).

Deep-inspiration breath-hold (DIBH) PET/CT with short-time acquisition and respiratory-gated (RG) PET/CT are performed to reduce the respiratory motion artifacts, and to obtain more accurate SUV [9–17].

The signal-to-noise ratio of RG PET is better because of the longer time of acquisition than that with DIBH PET. The RG PET/CT can also be used for patients who cannot maintain breath holding for a long duration. However, device preparation and acquisition time are somewhat cumbersome and take a longer time, which may cause a burden to the patients or delay in study schedule. RG CT has major drawbacks in terms of high radiation exposure because of repeated cine mode scan and poor image quality due to the body motion of free breathing and low tube current time product.

While the image quality of DIBH PET is generally inferior to that of RG PET due to short-time acquisition, DIBH PET/CT has significant advantages in terms of its rapid examination, better CT image quality and low radiation exposure.

In our study, RG PET has been regarded as a gold standard, and its detectability between DIBH and RG PET was compared using each of the most optimal reconstruction parameters demonstrated in the phantom study. To the best of our knowledge, this is the first report on lesions providing equivalent image quality between DIBH and RG PET. In addition, no study to date has been conducted to determine the reconstruction parameters of DIBH PET on the basis of the optimal reconstruction parameters of RG PET.

## Materials and methods

### Phantom study

**Fig. 1:**
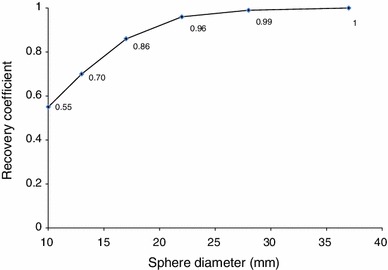
The recovery coefficient (RC) of the PET/CT system used in this study. The RCs were determined referring to the Japanese Guideline for Oncology PET/CT. The phantom image was reconstructed by the optimal reconstruction parameters for RG PET

The National Electrical Manufacturers Association (NEMA) 2001 International Electrotechnical Commission (IEC) phantom (Data Spectrum Corp., Hillsborough, NC) was used for this study. This phantom consisted of a torso cavity, a removable lung insert, and 6 spheres. The inner diameters of these spheres were 10, 13, 17, 22, 28 and 37 mm. They were filled with ^18^F-FDG solutions of the same radioactivity concentration (10.6 kBq/mL), and the background (BG) was set to 2.65, 1.33 kBq/mL and none (air). They were scanned using a list-mode dynamic acquisition method. The most optimal reconstruction parameters for RG PET were determined referring to the Japanese Guideline for Oncology of FDG-PET/CT [18], and the phantom filled with 2.65 kBq/mL in the BG was used. Other phantoms were used to determine the optimal reconstruction parameters for DIBH PET. At our institution, ^18^F-FDG is injected with radioactivity of 4.4 kBq/g, and RG and DIBH PET are performed at about 150 min after injection (physical decay to 39 %). If the percentage of injected radioactivity excreted to the bladder is 23 % [19], and the percentage of the adipose tissue is 27 % of the total body volume, the radioactivity of the mediastinum at 150 min after injection is estimated to be 1.81 kBq/mL (4.4 kBq/g × 1 g/mL × 0.39 × 0.77/0.73 = 1.81), which is equivalent to 1.05 SUV (0.77/0.73 = 1.05). Then, 1.33 kBq/mL is equivalent to 0.77 SUV. In addition, 10.6 kBq/mL is equivalent to 6.16 SUV, and the SUV of the 10-mm sphere is equivalent to 3.39 SUV because the recovery coefficient (RC) for the 10-mm sphere of the PET/CT system used in this study is 0.55 based on our preliminary examination (Fig. 1).

### Data acquisition

PET/CT scans were performed using Discovery PET/CT 600 Motion (GE Healthcare, Milwaukee, WI). Emission durations were set to 2 min and 20 s. The 2-min scanned images were simulated by RG PET because our clinical scan protocol involved 10-min acquisition and was divided into 5 bins. The 20-s scanned images were simulated by DIBH PET. All images were reconstructed using a 3-dimensional ordered subset-expectation maximization (3D-OSEM) algorithm with VUE point plus and Gaussian filter. The transaxial field of view (FOV) of the reconstructed image was 550 mm, the slice thickness was 3.27 mm, and the matrix size was 128 × 128.

The 16-slice CT scanning was performed using 120 kVp, 10–200 mA, noise index 10, rotation time 0.6 s, pitch 1.75:1 and slice thickness 3.75 mm. All CT images were reconstructed by transaxial FOV 500 mm, and the matrix size 512 × 512.

### Data analysis and image reconstruction

For visual analysis, the PET images were evaluated by three certificated PET technologists including one nuclear medicine expert, who were engaged in PET work for more than 5 years. The images were displayed using an inverse gray scale with a SUV range of 0–4. Each sphere was scored by five grades; very good image quality 5, sufficient good image quality 4, scarcely sufficient image quality 3, not sufficient image quality 2, and unreadable 1. When the visual score was ≥3, it was judged as the sphere was detectable.

For physical indexes, the simulated RG PET was reconstructed using iteration-subset combinations of 2-16, 3-16, 2-32, and 5-16. Full width at half maximum (FWHM) of the Gaussian filter was changed within 3.5–7 mm for each reconstruction parameter. The smallest detectable sphere (*X* mm) was used to measure the mean SUV (SUVmean_H,*X*mm_) using a region of interest (ROI) of the same diameter, and the center slice where the sphere was the most prominent was used. The BG was determined using 12 ROIs on the same slice, and the average of mean SUV (SUVmean_BG,*X*mm_) was calculated. The percent contrast (*Q*_H,*X*mm_) were calculated by:

$$Q_{{{\text{H,}}X{\text{mm}}}}\, =\, \frac{{{\text{SUVmean}}_{{{\text{H,}}X{\text{mm}}}} / {\text{SUVmean}}_{{{\text{BG,}}X{\text{mm}}}} - 1}}{4 - 1} \times\, 100\,(\%).$$

Further, to calculate the percent BG variability (*N*_*X*mm_), 12 ROIs were set on the slice and similarly on additional four slices (±1 and ±2 cm of the upper and lower sides from the center slice). The *N*_*X*mm_ was calculated by a total of 5 slices using average value of 60 ROIs (SUVmean_BG_,_*X*mm,60_) using the following formula:

$$N_{{X{\text{mm}}}} = \frac{{{\text{SD}}_{{X{\text{mm}}}} }}{{{\text{SUVmean}}_{{{\text{BG}},X{\text{mm}},60}} }} \times 100\;(\%).$$

SD_*X*mm_ was the standard deviation (SD) of the BG, calculated as follows:

$${\text{SD}}_{{X{\text{mm}}}} = \sqrt {\sum\limits_{k = 1}^{k} {({\text{SUVmean}}_{{{\text{BG,}}X{\text{mm,}}k}} - {\text{SUVmean}}_{{{\text{BG,}}X{\text{mm,}}60}} )^{2} /(k - 1)} } ,\quad k = 60.$$

Considering the statistical variation of PET images, *Q*_H,*X*mm_ and *N*_*X*mm_ were calculated based on the average of three images, which were reconstructed from 0, 1 and 2 min after the starting time.

With respect to the simulated DIBH PET, the images were reconstructed using iteration-subset combinations of 2-8, 2-16 and 2-32. The FWHM of the Gaussian filter was set to be 4.7 mm. The maximum SUV (SUVmax) and radioactivity (kBq/mL) of each sphere were measured using the ROI of the same diameters from the slice where these spheres were most obviously observed. A % difference in SUVmax (% Dif) was defined as follows: % Dif = simulated DIBH PET SUVmax/simulated RG PET SUVmax. The reconstruction parameters which showed similar visual and physical evaluations on the simulated RG PET were determined as the most optimal parameters for DIBH PET.

The average of each SUVmax and maximum radioactivity was calculated from the three slices which were reconstructed from the standard time and 1 and 2 min later.

### Clinical study

The most optimal reconstruction parameters for DIBH and RG PET were used to examine the clinical study. Nineteen patients with a pulmonary lesion (mean 18.5 ± 7.2 mm, range 10–32 mm) consisted of 12 males and 7 females (mean 68.8 ± 11.9 years, range 34–87 years) (Table 1). They were examined for staging of lung cancer or for being suspect of malignancy of the lung. All patients were free from chronic obstructive pulmonary disease.

**Table 1:** Characteristics of patients with pulmonary lesions

Patient	Age	Sex	Site	Maximum diameter (mm)
1	72	M	L lower lobe	10
2	69	M	L lower lobe	10
3	76	F	R upper lobe	10
4	70	M	L lower lobe	10
5	76	M	L lower lobe	11
6	67	M	L lower lobe	12
7	72	F	L upper lobe	14
8	59	M	R lower lobe	15
9	34	M	L upper lobe	16
10	60	F	R lower lobe	20
11	61	F	R lower lobe	20
12	86	F	L lower lobe	20
13	70	M	R lower lobe	22
14	87	M	R upper lobe	23
15	59	M	L upper lobe	25
16	71	M	R upper lobe	25
17	62	F	L upper lobe	26
18	79	F	R middle lobe	30
19	78	M	L lower lobe	32

*M* male, *F* female, *L* left, *R* right

After all patients fasted at least 5 h, ^18^F-FDG was injected with radioactivity of 4.4 MBq/kg (maximum dose 330 MBq). The RG and DIBH PET were performed at 143 ± 11, and 156 ± 11 min, respectively, after injection.

In RG PET/CT study, the respiratory motion of patients was recorded by a respiratory gating device (Varian Medical Systems, Palo Alto, CA) during the CT and PET scanning. RG CT was scanned using a cine mode, and the scan time was a breathing cycle time plus about 1 s. This scan was repeated eight times to include an axial FOV of 154 mm for the PET system. The interval time between image reconstructions was set to 0.5 s. Emission data were acquired for 10 min using the list-mode dynamic acquisition method. The respiratory cycles were divided into 5 bins. The adequate bin determined to have the highest SUVmax of the lesions was chosen for RG PET SUVmax. The RG CT scanning parameters were 120 kVp, 10–100 mA, noise index 35, rotation time 0.5 s, and slice thickness 2.5 mm.

In DIBH PET/CT study, after the 3-s CT scans, one bed PET acquisition for 20 s was repeated three times. For both the CT and PET acquisitions, no chest wall movement was confirmed by monitoring respiratory gating device, which indicated no misregistration between the two modalities. Among the three repeated acquisitions, the most adequate acquisition showing the highest SUVmax of the lesions was chosen for DIBH PET SUVmax. The DIBH CT scanning parameters were 120 kVp, 10–200 mA, noise index 30, rotation time 0.6 s, pitch 1.75:1, and slice thickness 3.75 mm.

All patients provided written informed consent. Both RG and DIBH PET/CT methods were routinely performed in our institute and not intended for research. All the data were anonymized and analyzed retrospectively, and the study was approved by the security policy of the hospital.

### Statistical analysis

All the data were shown using mean and SD. In the phantom study, significant differences were examined using Tukey’s method. The levels of significance were set at <0.05. In the clinical study, correlation and Bland–Altman analyses were performed using the SUVmax of the both methods [20]. The difference in SUVmax between RG and DIBH PET was calculated, and the 95 % limit of agreement was calculated by mean ± 1.96 SD.

## Result

### The most optimal reconstruction parameters for RG PET

The visual scores of hot areas regarding reconstruction parameters are shown in Table 2. Visual scores were independent from iteration-subset combinations and FWHM of the Gaussian filter if the sphere diameters were the same. When the sphere diameters were 10 and 13 mm, the visual scores were <3, indicating that the spheres were not detected. Since the score of 17 mm of the sphere was over 4 regardless of the reconstruction parameters, the smallest detectable sphere was the 17 mm one.

**Table 2:** Visual scores of hot areas regarding reconstruction parameters

Iteration-subset	FWHM (mm)	Sphere diameters (mm)					
		10	13	17	22	28	37
2-16	3.5	2.1 ± 1.1	2.3 ± 1.2	4.4 ± 0.5	5.0	5.0	5.0
	4.7	2.1 ± 1.1	2.4 ± 1.2	4.4 ± 0.5	5.0	5.0	5.0
	5.9	1.9 ± 1.2	2.4 ± 1.0	4.3 ± 0.5	5.0	5.0	5.0
	7	1.4 ± 0.7	2.6 ± 0.7	4.2 ± 0.4	4.9 ± 0.3	5.0	5.0
3-16	3.5	2.1 ± 1.2	2.8 ± 1.1	4.3 ± 0.5	4.8 ± 0.4	5.0	5.0
	4.7	2.2 ± 1.2	2.7 ± 1.0	4.4 ± 0.5	4.8 ± 0.4	5.0	5.0
	5.9	2.0 ± 1.1	2.8 ± 1.2	4.4 ± 0.5	4.9 ± 0.3	5.0	5.0
	7	2.0 ± 1.0	2.8 ± 0.8	4.3 ± 0.5	4.8 ± 0.4	5.0	5.0
2-32	3.5	2.1 ± 1.5	2.6 ± 1.2	4.4 ± 0.5	4.8 ± 0.4	5.0	5.0
	4.7	2.1 ± 1.2	2.8 ± 1.0	4.6 ± 0.5	4.8 ± 0.4	5.0	5.0
	5.9	2.0 ± 1.1	2.9 ± 1.2	4.6 ± 0.5	4.9 ± 0.3	5.0	5.0
	7	1.9 ± 1.2	2.7 ± 0.9	4.4 ± 0.5	4.9 ± 0.3	5.0	5.0
5-16	3.5	2.2 ± 1.5	2.8 ± 1.4	4.4 ± 0.5	4.8 ± 0.4	5.0	5.0
	4.7	2.0 ± 1.2	2.8 ± 1.2	4.4 ± 0.5	4.8 ± 0.4	5.0	5.0
	5.9	2.1 ± 1.1	2.8 ± 0.8	4.4 ± 0.5	4.9 ± 0.3	5.0	5.0
	7	1.9 ± 0.9	2.9 ± 0.9	4.6 ± 0.5	4.9 ± 0.3	5.0	5.0

*FWHM* full width at half maximum

The physical indexes were evaluated as shown in Table 3. The mean *Q*_H,17mm_ of 2-32 and 5-16 was 54.8 ± 3.2 and 54.7 ± 4.9 %, respectively. These parameters were significantly higher than those of 2-16 and 3-16 (*p* < 0.01). The mean *N*_17mm_ of 2-32 and 5-16 was 7.4 ± 1.0 and 8.2 ± 0.6 %, respectively. The *N*_17mm_ of 2-32 was significantly inferior to that of 2-16 (*p* < 0.01) and 3-16 (*p* < 0.05), and the *N*_17mm_ of 5-16 was significantly inferior to that of 2-16 and 3-16 (*p* < 0.01). The mean *Q*_H,17mm_/*N*_17mm_ of 5-16 was 6.7 ± 0.1 %, and it was significantly lower than those of other parameters (*p* < 0.01). Based on these results, the optimal iteration-subset combination was determined as 2-32. For the FWHM of the Gaussian filter, the *Q*_H,17mm_ and *N*_17mm_ of 2-32 did not differ significantly regardless of the FWHM of the Gaussian filter except for the *Q*_H,17mm_ between 3.5 and 7-mm FWHM (*p* < 0.05).

**Table 3:** Physical indexes based on reconstruction parameters

Physical index	Iteration -subset	FWHM (mm)				Mean
		3.5	4.7	5.9	7	
*Q*_H,17mm_ (%)	2-16	48.0 ± 1.8	45.5 ± 3.5	44.6 ± 1.4	41.1 ± 1.4	44.8 ± 3.2
	3-16	51.7 ± 4.5	49.2 ± 4.6	47.1 ± 2.8	46.0 ± 4.7	48.5 ± 4.3
	2-32	58.6 ± 2.2	54.9 ± 2.5	54.0 ± 1.4	51.7 ± 2.1	54.8 ± 3.2*
	5-16	58.6 ± 5.0	56.3 ± 5.0	52.5 ± 4.1	51.1 ± 3.7	54.7 ± 4.9**
*N*_17mm_ (%)	2-16	6.6 ± 0.6	6.3 ± 0.4	6.1 ± 0.5	5.8 ± 0.6	6.2 ± 0.6
	3-16	7.0 ± 1.3	6.5 ± 1.3	6.2 ± 1.0	5.9 ± 1.0	6.4 ± 1.1
	2-32	7.9 ± 1.0	7.5 ± 1.3	7.3 ± 1.1	7.0 ± 1.0	7.4 ± 1.0^†^
	5-16	8.7 ± 0.8	8.4 ± 0.6	7.9 ± 0.4	7.7 ± 0.3	8.2 ± 0.6^††^
*Q*_H,17mm_/*N*_17mm_	2-16	7.2 ± 0.8	7.3 ± 0.7	7.3 ± 0.7	7.1 ± 0.8	7.2 ± 0.1
	3-16	7.4 ± 1.5	7.5 ± 1.7	7.6 ± 1.3	7.8 ± 1.6	7.6 ± 0.2
	2-32	7.5 ± 1.0	7.4 ± 1.3	7.4 ± 1.1	7.4 ± 1.1	7.4 ± 0.0
	5-16	6.8 ± 0.8	6.7 ± 0.8	6.6 ± 0.6	6.6 ± 0.6	6.7 ± 0.1^§^

*FWHM* full width at half maximum

* The mean *Q*_H,17mm_ of the 2-32 was significantly higher than those of 2-16 and 3-16 (*p* < 0.01)

** The mean *Q*_H,17mm_ of the 5-16 was significantly higher than those of 2-16 and 3-16 (*p* < 0.01)

^†^The mean *N*_17mm_ of the 2-32 was significantly higher than those of 2-16 (*p* < 0.01) and 3-16 (*p* < 0.05)

^††^The mean *N*_17mm_ of the 5-16 was significantly higher than those of 2-16 and 3-16 (*p* < 0.01)

^§^The mean *Q*_H,17mm_/*N*_17mm_. of the 5-16 was significantly lower than those of 2-16, 3-16 and 2-32 (*p* < 0.01)

**Fig. 2:**
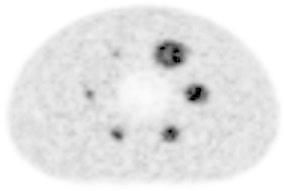
The phantom image reconstructed by the iteration-subset combination of 2-32 and 4.7-mm FWHM of the Gaussian filter

The phantom image based on the iteration-subset combination of 2-32 and 4.7-mm FWHM of the Gaussian filter is shown in Fig. 2. Both hot areas and a cold area in the center are clearly visualized, although hot areas of 10 and 13 mm were judged as suboptimal quality.

### The most optimal reconstruction parameters for DIBH PET

The visual scores are shown in Tables 4 and 5. When the BG was filled with 1.33 kBq/mL of water, the 10- and 13-mm spheres of the simulated DIBH PET could not be detected regardless of the reconstruction parameters. The 17-mm sphere was, however, detectable depending on the reconstruction parameters (2-8 and 2-16) although the scores were significantly lower than that of the simulated RG PET (*p* < 0.05 and 0.01). On the other hand, when the BG was filled with air, even the 10-mm sphere of the DIBH PET had sufficient image quality for all reconstruction parameters, and the visual scores were comparable to that of the RG PET.

**Table 4:** Visual scores of the phantom filled with radioactivity of 1.33 kBq/mL in the BG

Simulated image	Iteration-subset	Sphere diameters (mm)					
		10	13	17	22	28	37
RG	2-32	4.0 ± 1.1	4.2 ± 1.2	4.7 ± 0.7	5	5	5
DIBH	2-8	1.4 ± 0.7	2.2 ± 0.7	3.7 ± 0.5**	4.7 ± 0.5	4.9 ± 0.3	5.0
	2-16	1.7 ± 0.9	2.4 ± 0.5	3.6 ± 0.5*	4.2 ± 0.7	4.7 ± 0.5	5.0
	2-32	1.3 ± 0.5	1.9 ± 0.6	2.8 ± 0.8*	3.9 ± 0.9**	4.3 ± 0.7	4.9 ± 0.3

All images were reconstructed by the 4.7-mm FWHM of the Gaussian filter. The 10 and 13-mm spheres cf simulated DIBH PET could not be detected regardless of the reconstruction parameters

*RG* simulated RG PET scanned for 2 min, *DIBH* simulated DIBH PET scanned for 20 s

The score of simulated DIBH PET was significantly lower than that of simulated RG PET (*p < 0.01, **p < 0.05)

**Table 5:** Visual scores of the phantom filled with air in the BG

Simulated image	Iteration-subset	Sphere diameters (mm)					
		10	13	17	22	28	37
RG	2-32	5	5	5	5	5	5
DIBH	2-8	4.8 ± 0.4	5.0	5.0	5.0	5.0	5.0
	2-16	4.9 ± 0.3	5.0	5.0	5.0	5.0	5.0
	2-32	4.9 ± 0.3	5.0	5.0	5.0	5.0	5.0

All images were reconstructed by the 4.7-mm FWHM of the Gaussian filter

*RG* simulated RG PET scanned for 2 min, *DIBH* simulated DIBH PET scanned for 20 s

Physical indexes were evaluated using the detectable spheres (Tables 6, 7). When the simulated DIBH PET was reconstructed by iteration-subset combinations of 2-8 and 2-16, significant differences were not confirmed between the maximum radioactivity of the DIBH PET and that of the RG PET regardless of the BG radioactivity and sphere diameters. However, when it was reconstructed by 2-32, significant differences were confirmed for some spheres.

**Table 6:** Physical indexes for the phantom filled with radioactivity of 1.33 kBq/mL in the BG

Simulated image	Iteration-subset	Parameter	Sphere diameters (mm)						Mean
			10	13	17	22	28	37	
RG	2-32	Max (kBq/mL)			9.7 ± 1.3	12.0 ± 0.6	11.9 ± 0.4	12.8 ± 0.3	
DIBH	2-8	Max (kBq/mL)			7.3 ± 0.6	10.1 ± 0.5	10.3 ± 0.5	13.2 ± 0.7	
		% Dif			74.4 ± 11.8	85.4 ± 5.8	86.2 ± 5.6	102.4 ± 6.3	87.1 ± 11.5
	2-16	Max (kBq/mL)	–	–	10.3 ± 0.9	12.5 ± 1.4	12.4 ± 0.8	14.8 ± 0.4	
		% Dif			106.4 ± 17.0	104.9 ± 13.1	103.7 ± 7.4	115.6 ± 4.4	107.6 ± 5.4^†^
	2-32	Max (kBq/mL)			13.8 ± 1.8**	14.9 ± 2.8	15.0 ± 1.6**	16.8 ± 1.3*	
		% Dif			142.5 ± 26.4	125.4 ± 24.5	125.3 ± 14.4	131.1 ± 10.7	131.1 ± 8.1^††^

All images were reconstructed by the 4.7-mm FWHM of the Gaussian filter

*RG* simulated RG PET scanned for 2 min, *DIBH* simulated DIBH PET scanned for 20 s, *Max* maximum radioactivity (kBq/mL), % *Dif* simulated DIBH PET SUVmax/simulated RG PET SUVmax × 100 (%)

The Max of simulated DIBH PET was significantly higher than that of simulated RG PET (*p < 0.01, **p < 0.05)

^†^The mean % Dif of 2-16 was significantly higher than that of 2-8 (*p* < 0.05)

^††^The mean % Dif of 2-32 was significantly higher than that of 2-16 (*p* < 0.05)

**Table 7:** Physical indexes for the phantom filled with air in the BG

Simulated image	Iteration-subset	Parameter	Sphere diameters (mm)						Mean
			10	13	17	22	28	37	
RG	2-32	Max (kBq/mL)	7.0 ± 0.8	9.6 ± 0.4	9.9 ± 0.4	10.4 ± 0.1	11.1 ± 0.3	11.5 ± 0.4	
DIBH	2-8	Max (kBq/mL) % Dif	6.4 ± 0.3	9.8 ± 0.3	10.4 ± 0.1	10.9 ± 0.1	11.3 ± 0.3	11.7 ± 0.4	101.0 ± 5.3
			90.8 ± 9.7	101.9 ± 5.5	105.8 ± 3.9	104.5 ± 2.0	102.3 ± 3.2	100.9 ± 5.2	
	2-16	Max (kBq/mL) % Dif	6.6 ± 0.6	9.4 ± 0.9	10.0 ± 0.2	10.9 ± 0.3	11.6 ± 0.5	11.9 ± 0.3	101.1 ± 4.5
			93.8 ± 12.8	97.8 ± 10.3	101.8 ± 4.1	104.8 ± 3.2	105.2 ± 4.9	103.1 ± 4.4	
	2-32	Max (kBq/mL) % Dif	6.8 ± 0.5	9.7 ± 1.0	9.5 ± 0.2	11.3 ± 0.6	11.8 ± 0.3	12.5 ± 0.2**	103.1 ± 5.8
			97.4 ± 12.1	100.4 ± 11.3	96.0 ± 3.8	108.3 ± 6.0	107.2 ± 3.0	109.1 ± 4.1	

All images were reconstructed by the 4.7-mm FWHM of the Gaussian filter

*RG* simulated RG PET scanned for 2 min, *DIBH* simulated DIBH PET scanned for 20 s, *Max* maximum radioactivity (kBq/mL), % *Dif* simulated DIBH PET SUVmax/simulated RG PET SUVmax × 100 (%)

The Max of simulated DIBH PET was significantly higher than that of simulated RG PET (**p < 0.05)

For the % Dif, when the phantom was filled with air in the BG, the mean % Dif of iteration-subset combinations of 2-8, 2-16 and 2-32 was 101.0 ± 5.3, 101.1 ± 4.5 and 103.1 ± 5.8 %, respectively (*p* = n.s.). On the other hand, when the phantom was filled with 1.33 kBq/mL in the BG, that of 2-8, 2-16 and 2-32 was 87.1 ± 11.5, 107.6 ± 5.4 and 131.1 ± 8.1 %, respectively. The % Dif of 2-16 was significantly higher than that of 2-8 (*p* < 0.05), and was significantly lower than that of 2-32 (*p* < 0.05). Based on these results, the most optimal reconstruction parameters for DIBH PET were 2-16.

### Clinical study

**Fig. 3:**
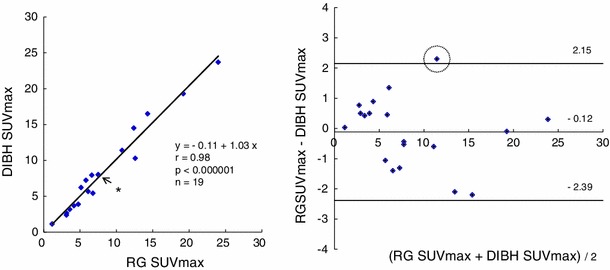
Correlation relationship (*left*) and Bland–Altman analysis (*right*) between RG and DIBH PET SUVmax. High correlation and little dispersion were observed. *This dot consisted of two lesions having very similar SUVmax. A case enclosed by the circle in the Bland–Altman analysis is shown in Fig. 6

The mean radiation doses that patients received from DIBH and RG CT were 0.60 and 7.10 mSv, respectively. Correlation and Bland–Altman analyses are shown in Fig. 3. A regression line of the lesions was calculated as *y* = −0.11 + 1.03*x*, *r* = 0.98, *p* < 0.000001. For Bland–Altman analysis, the mean of RG PET SUVmax − DIBH PET SUVmax was −0.12, and the mean ± 1.96 SD ranged from −2.39 to 2.15. Eighteen patients were within the mean ± 1.96 SD.

**Fig. 4:**
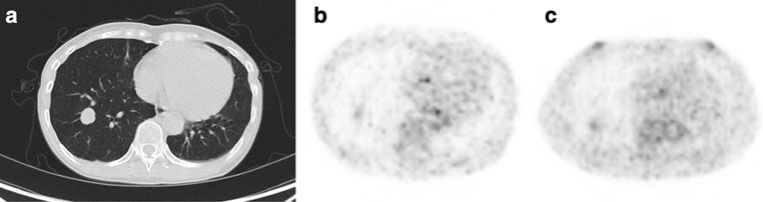
A 60-year-old female patient with an isolated pulmonary lesion in the right lower lobe. DIBH CT (**a**), DIBH PET (**b**), and RG PET (**c**)

**Fig. 5:**
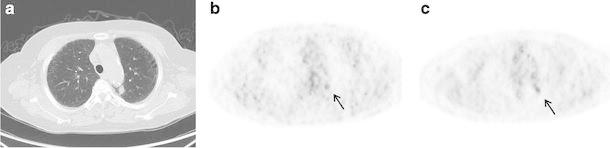
A 72-year-old female patient with a pulmonary lesion located close to the mediastinum in the left upper lobe (*arrow*). DIBH CT (**a**), DIBH PET (**b**), and RG PET (**c**)

**Fig. 6:**
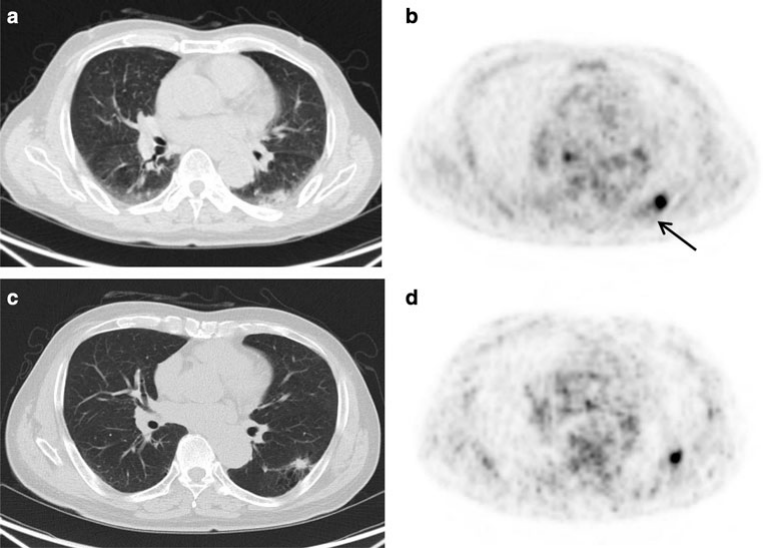
A 76-year-old male patient with a pulmonary lesion in the left lower lobe. RG CT (**a**), RG PET (**b**), DIBH CT (**c**), and DIBH PET (**d**). The RG PET SUVmax was increased by the poorly inflated dorsal lung with relatively high radioactivity (*arrow*)

Figure 4 shows an isolated pulmonary lesion of 20 mm in diameter. The DIBH and RG PET SUVmax were 1.14 and 1.17, respectively. Figure 5 shows a pulmonary lesion of 14 mm in diameter located close to the mediastinum. The DIBH and RG PET SUVmax were 2.66 and 3.16, respectively. These lesions could be observed not only by RG PET but also by DIBH PET. Figure 6 shows a pulmonary lesion of 11 mm in diameter enclosed by the circle in Fig. 3. The SUVmax of DIBH and RG PET were 10.3 and 12.6, respectively.

## Discussion

DIBH and RG PET/CT are widely performed to reduce respiratory motion artifacts. For the PET image, the image quality of DIBH PET is generally inferior to that of RG PET due to short-time acquisition. The image quality of RG PET is, however, better because of the longer time acquisition compared with that of DIBH PET. For the CT image, the image quality of DIBH CT is much superior to that of RG CT because the CT is acquired at the maximum inspiration position, which provides clear delineation like clinical CT routinely performed. The image quality of RG CT is, however, inferior to that of DIBH CT caused by the body motion of free breathing and low tube current time product. DIBH PET/CT has significant advantages in terms of rapid examination and low radiation exposure. While about 15 min of RG PET/CT examination was needed, <5 min was necessary for DIBH PET/CT. In terms of radiation doses, RG CT was about 12 times higher than that of DIBH CT. Therefore, we compared the detectability between DIBH and RG PET, and the limitation and indication for DIBH PET were evaluated.

For DIBH PET acquisition time, Miyashita et al. [17] reported that optimum emission time of the DIBH PET technique greater than 90 s acquisition is preferable for clinical use. However, the optimal acquisition time varied according to PET systems, and a long acquisition time and repeated acquisition were not acceptable for patients with pulmonary lesions as Kawano et al. [13] noted in a clinical setting that using DIBH PET with a breath holding of <30 s could be helpful. The PET system used in this study has high sensitivity (3D: 9.1 cps/kBq). Torizuka et al. [12] have reported that a single 20 s acquisition of breath-hold PET/CT enabled more precise measurement of tumor ^18^F-FDG uptake. DIBH PET acquisition time was, therefore, set to be 20 s, which might be the clinical upper limit of breath holding.

The most optimal reconstruction parameters for DIBH and RG PET were determined in the phantom study, and those of RG PET were regarded as a gold standard. The *Q*_H,17mm_ was more important than the *N*_17mm_ because most of the pulmonary lesions were surrounded by low BG radioactivity. The optimal iteration-subset combination was found to be 2-32 based on high *Q*_H,17mm_ and *Q*_H,17mm_/N_17mm_. To determine the FWHM of the Gaussian filter, since the *Q*_H,17mm_ and *N*_17mm_ of 2-32 did not differ significantly among filter types, we selected 4.7-mm FWHM in accordance with our clinical parameter. Pulmonary lesions located close to the mediastinum were simulated using the phantom filled with 1.33 kBq/mL in the BG, which is similar to clinical conditions. The lesions surrounded by pulmonary region were simulated using the phantom filled with air in the BG. Even the 10-mm sphere was clearly visualized when those phantom images were reconstructed by this parameters. Consequently, it was judged that the reconstruction parameters, acquisition time and number of bins for RG PET were appropriate.

The optimal reconstruction parameters of DIBH PET were different from that of RG PET, and reconstruction parameters of DIBH PET could be obtained by reducing the number of subsets for those of RG PET in the state of fixing the number of iterations. The reason for this was that the images with low count such as DIBH PET may have been diverged when the number of subsets was high [21]. Using the same reconstruction parameters as that of RG PET, the maximum radioactivity of the simulated DIBH PET was significantly higher for some spheres compared to that of the simulated RG PET.

The clinical study was performed using each of the most optimal reconstruction parameters. In 19 cases, high correlation and little dispersion were observed between DIBH and RG PET SUVmax. In our study, since most of the lesions were surrounded by low pulmonary radioactivity, the overlapped radioactivity from the mediastinum was negligible. This result was consistent with that of the phantom study filled with air in the BG. Even the 10-mm sphere showed equivalent image quality between the simulated DIBH PET and RG PET.

The DIBH PET showed high contrast between the lesion and the BG because the lung was filled with a significant amount of air for maximum inspiration during breath holding. The lesion was visible as low as SUVmax of 1.1, and DIBH and RG PET SUVmax had nearly equivalent values. When the lesion was located close to the mediastinum, DIBH and RG PET also had nearly equivalent image quality since the major part of the lesion was surrounded by low pulmonary radioactivity. In Fig. 6, the high SUVmax was obtained even in a small lesion. Several articles have reported that the SUVmax of the lesions which are small and located in the lower lung is especially decreased by the respiratory motion artifact under free breathing [11–13]. Since the lesion in this patient was also small and in the lower lobe, the underestimation of SUVmax was highly improved by DIBH and RG PET/CT. However, the difference between DIBH and RG PET SUVmax was the highest in the clinical study. The RG PET SUVmax was overestimated because the lesion was surrounded by poorly inflated dorsal lung with relatively high radioactivity (SUVmean 1.76). Using the DIBH PET/CT, the dorsal vascular shadow was not found. This case has shown that DIBH PET could provide higher accurate SUVmax than that of RG PET. Regarding CT image quality, the DIBH CT could clearly describe the lesion, but the RG CT could not.

This study has limitations. As shown in the phantom study filled with 1.33 kBq/mL in the BG, the sphere size <17 mm could not be detected. The equivalent image quality between DIBH and RG PET might not be obtained according to the BG radioactivity around the lesion. The detectability of lesions located close to the mediastinum could be limited depending on their sizes and accumulations. All of the lesions surrounded by the pulmonary region may not provide equivalent image quality between DIBH and RG PET as shown in Fig. 6. Further clinical assessment is indicated in this respect.

DIBH PET/CT has a significant practical value, but poor signal-to-noise ratio caused by the size, uptake and target to BG contrast of the lesions. RG PET/CT and DIBH PET/CT under multiple summed acquisition methods reported by Nehmeh et al. [14–17] are preferable to assess the lesions which are difficult for detecting using DIBH PET.

## Conclusion

DIBH PET/CT may be the most practical method which can be the first choice to reduce respiratory motion artifacts if the detectability of DIBH PET is equivalent with that of RG PET. Although DIBH PET may have limitations in suboptimal signal-to-noise ratio due to the short-time acquisition, most of the lesions surrounded by low BG radioactivity could provide nearly equivalent image quality between DIBH and RG PET studies when each of the most optimal reconstruction parameters was used.
